# GFAP Use in Emergency Medicine—A Narrative Review

**DOI:** 10.3390/neurosci7050108

**Published:** 2026-09-30

**Authors:** Mohamed Tharwat Ghazala, Idris Sula, Muhammad Candragupta Jihwaprani, Amjaad Haban Aljabri, Sohaib Yaser Saif, Ahmed Alhebshi, Hussam Ghassub Alatae, Ahmed El-Sayed Hassan

**Affiliations:** 1 Department of Clinical Sciences, College of Medicine, Sulaiman Al Rajhi University, Al Bukayriyah 51941, Saudi Arabia; 2 Department of Medical Laboratory Sciences, College of Applied Sciences, Sulaiman Al Rajhi University, Al Bukayriyah 52726, Saudi Arabia; 3 Department of Medical Education, Sidra Medicine, Doha P.O. Box 26999, Qatar; 4 Department of Basic Sciences, College of Medicine, Sulaiman Al Rajhi University, Al Bukayriyah 51941, Saudi Arabia; 5 Department of Medical Physiology, College of Medicine, Zagazig University, Zagazig 44519, Egypt

**Keywords:** GFAP, biomarkers in emergency medicine, CNS injury, neurological emergencies, traumatic brain injury (TBI), astrocytes

## Abstract

Background: Traditional clinical assessments for neurological emergencies, such as the Glasgow Coma Scale, are often subjective and may be confounded by factors like intoxication or sedation. While computed tomography (CT) remains the diagnostic standard, it may lack sensitivity for mild traumatic brain injury (TBI) and pose a risk of radiation exposure. Glial fibrillary acidic protein (GFAP), an intermediate filament protein primarily produced by astrocytes in the central nervous system (CNS), has been shown to be a potential objective marker of neuronal distress, even when macrostructural imaging may appear normal. Aims: This narrative review synthesizes recent evidence on the molecular biology and pathophysiology of GFAP in acute CNS injury. It evaluates GFAP’s clinical applications across major neurological emergencies—including TBI, stroke, and sepsis—while comparing its performance to other major biomarkers and identifying the technological and operational hurdles for routine emergency department (ED) implementation. Evidence Synthesis: Following CNS insult, astrocytes undergo reactive astrogliosis, leading to GFAP upregulation and proteolytic cleavage into breakdown products (GFAP-BDP) that provide a unique signature of structural disruption. GFAP exhibits favorable kinetics in the circulation, with a rise within one hour and a peak at approximately 20 h. In TBI, GFAP is FDA-cleared for CT decision-making and demonstrates superior diagnostic accuracy (AUC 0.88–0.89) within the first 30–60 min of injury. Some studies also support its role in distinguishing intracerebral hemorrhage from ischemic stroke according to distinct temporal release patterns and serve as a potential biological marker of CNS involvement in sepsis-associated encephalopathy; though its use is still investigational and requires further validation. Compared with other biomarkers such as S100B, UCH-L1, NSE, and tau, GFAP offers greater CNS specificity and more reliable signals for identifying structural lesions. Conclusions: GFAP represents a transformation tool for objective risk stratification and decision-making in the ED. However, its transition from a research tool to a standard clinical pillar requires the standardization of assay platforms, established reference materials, and large-scale validation of clinical thresholds.

## 1. Introduction

### 1.1. Overview of Biomarkers in Emergency Medicine

Since the introduction of creatine kinase–MB (CK-MB) as a diagnostic marker for myocardial injury in the early 1970s [1], biomarkers have revolutionized the diagnosis and risk stratification of acute conditions, laying the groundwork for the modern expansion of biomarker use in emergency medicine. Contemporary emergency practice integrates both central laboratory assays and point-of-care testing (POCT), the latter enabling real-time bedside decision-making that shortens diagnostic delays, enhances patient flow, and improves overall quality of care [2,3]. Widely used biomarkers such as troponin, D-dimer, and brain natriuretic peptide (BNP)/N-terminal proBNP (NT-proBNP) remain foundational in Emergency Department (ED) diagnostic pathways [4,5,6], while emerging markers such as interleukin-6, presepsin, glial fibrillary acidic protein (GFAP), neuron-specific enolase, and S100B show promise for early detection of sepsis and neuronal injury [7,8,9]. These trends underscore the evolving landscape in which biomarkers are central to precise and accelerated diagnostics in crowded emergency care settings.

### 1.2. Importance of Rapid, Objective Markers for CNS Injury and Neurological Emergencies

In neurological emergencies, “Time is Brain.” For every minute a stroke or severe traumatic brain injury (TBI) goes untreated, irreversible changes are being established [10]. Objective markers have the potential to provide several critical advantages, such as overcoming subjectivity and the capability to rule out with high sensitivity. Tools like the Glasgow Coma Scale (GCS) depend on a clinician’s observation and a patient’s ability to respond [11]. On the other hand, markers such as GFAP provide a molecular snapshot that does not rely on whether a patient is conscious or intoxicated [12]. Biomarkers can detect cellular distress even when the brain’s macrostructure looks normal. While CT could show nothing in mild TBI, it prevents patients from being discharged with hidden injuries [13].

### 1.3. Introduction to GFAP

GFAP is an intermediate filament protein primarily expressed in central nervous system (CNS) astrocytes, where it acts as a key structural scaffold for these cells [14]. The GFAP monomer is approximately 432 amino acids long and has three distinct domains: an N-terminal head domain, a central α-helical rod domain, and a small C-terminal tail domain [12]. GFAP monomers assemble into cytoskeletal filaments that provide structural support to astrocytes. Two GFAP monomers first form a parallel homodimer, and in turn, two dimers align anti-parallel to create a staggered tetramer. Subsequent lateral and end-to-end association of tetramers generates the mature intermediate filaments that strengthen the astrocytic cytoskeleton. This hierarchical assembly of GFAP contributes to the integrity and resilience of astrocytes under mechanical and metabolic stress [12].

In normal physiology, GFAP expression increases as astrocytes mature, and its gene transcription can be modulated by various signals [15,16,17]. GFAP plays a pivotal role in the central nervous system’s response to injury and disease. It is markedly upregulated during reactive astrogliosis—the process in which astrocytes proliferate and hypertrophy in response to CNS insults such as trauma, ischemia, or inflammation [18]. Astrocytic activation begins within minutes to hours after injury and typically peaks within 24–72 h [18]. This response is initiated through multiple mechanisms [19]. The accumulation of GFAP in reactive astrocytes is a hallmark of glial scar formation, reflecting its importance in restoring CNS structural integrity after injury. GFAP is also extensively post-translationally modified in these contexts, most notably through phosphorylation, which can alter filament assembly and astrocyte function [20]. Such dynamic changes in GFAP content and structure influence astrocyte morphology and the surrounding tissue environment, underscoring the protein’s clinical significance as both a marker of astrocytic activation and a contributor to neuropathology. It is also important to highlight that intracellular GFAP expression within astrocytes and measurable GFAP in CSF or blood should not be interpreted as biologically equivalent. Circulating GFAP reflects a composite signal that is influenced by astrocytic activation, proteolysis, blood–brain barrier (BBB) permeability, CSF or glymphatic drainage, assay characteristics, and clearance kinetics.

### 1.4. Rationale for a Narrative Review: Synthesizing Recent Advances and Highlighting Translational Potential

The field of GFAP research in clinical neurology has rapidly evolved over the past few years, with multiple prospective clinical studies, diagnostic validation trials, and mechanistic investigations demonstrating the utility of GFAP as a biomarker across diverse acute neurological emergencies [15]. In 2020, the United States Food and Drug Administration (US-FDA) cleared the first point-of-care (POC) GFAP assay (Roche Elecsys^®^ GFAP on cobas e801 (Mannheim, Germany), later followed by Abbott Alinity^®^ (Abbott Park, IL, USA) and Lumipulse^®^ platforms (Tokyo, Japan)) for the determination of the need for head computed tomography within 12 h after mild traumatic brain injury (TBI; GCS of 13–15) [16,17]. Despite this regulatory progress, GFAP remains underutilized in many emergency departments, and awareness of its applications beyond mild TBI is limited among emergency physicians. Recent 2024–2025 publications have substantially expanded the evidence base for GFAP across multiple acute neurological conditions, including severe TBI, acute ischemic and hemorrhagic strokes, cardiac arrest with hypoxic–ischemic encephalopathy (HIE), and CNS infections [18,19].

This review, in comparison to other reviews on GFAP, differs in few aspects. For instance, many reviews on GFAP focus on a particular aspect of GFAP such as addressing the role of GFAP in the early diagnosis and prognosis assessment of dementia, or as a marker to rule out intracranial lesions after TBI, or its potential in cerebral malaria, etc. [21,22,23]. The current review brings a broader evaluation of the uses of GFAP in emergency medicine. The main aims of this review are to (1) synthesize recent evidence on the molecular biology and pathophysiology of GFAP in acute CNS injury; (2) comprehensively review GFAP’s clinical applications and diagnostic performance across major neurological emergencies encountered in emergency medicine practice; (3) discuss the advantages and limitations of GFAP as a diagnostic and prognostic biomarker; (4) highlight technological advances in GFAP measurement platforms; and (5) identify knowledge gaps and future research directions to accelerate GFAP’s translation into routine emergency clinical practice [24].

## 2. Molecular Biology and Pathophysiology of GFAP

### 2.1. Structure and Assembly

GFAP is an intermediate filament protein whose structure has been extensively characterized through computational modeling and biochemical studies [14]. The protein comprises 432 amino acids in the predominant GFAP-α isoform. Its structure is organized into three distinct domains: (1) the N-terminal head domain (residues 1–81), which is intrinsically disordered and contains multiple phosphorylation sites; (2) the rod domain (residues 82–430), containing four α-helical coils (1A, 1B, 2A, 2B) separated by three linker regions (L1, L12, L2); and (3) the C-terminal tail domain, which varies among isoforms [12].

The rod domain is the most conserved region across intermediate filament proteins and is essential for filament assembly. GFAP monomers assemble into parallel homodimers in an antiparallel orientation, which then associate into tetramers, octamers, and longer filaments with a diameter of approximately 10 nanometers [25].

### 2.2. Regulation of GFAP Expression

GFAP expression is regulated at multiple levels [26]:

Transcriptional Regulation: GFAP transcription is activated by various nuclear receptor hormones (thyroid hormone, glucocorticoids), growth factors (ciliary neurotrophic factor [CNTF], fibroblast growth factor [FGF], transforming growth factor-β1 [TGF-β1]), bacterial endotoxins (lipopolysaccharides [LPS]), and pro-inflammatory cytokines [27]. Toll-like receptor (TLR) signaling, particularly TLR4 activation by LPS, is a major driver of GFAP upregulation in astrocytes and contributes to neuro-inflammation in response to bacterial infection and systemic sepsis [28,29]. GFAP undergoes several post-translational modifications that regulate its function by multiple protein kinases (protein kinase A, calmodulin-dependent kinase II, protein kinase C) that phosphorylate GFAP at N-terminal sites (T7, S8, S13, S17, S38, and S289). Phosphorylation generally inhibits GFAP filament assembly and is elevated following hypoxic–ischemic injury [30]. Following traumatic and ischemic brain injury, GFAP is cleaved by calpain (a calcium-activated protease) at sites N59-A60 and T383-F384, producing breakdown products (GFAP-BDP) of 38–44 kilodaltons. The resulting “limit fragment” lacks both N- and C-terminal domains but retains the intact rod domain. Caspase proteases also contribute to GFAP cleavage in pro-apoptotic conditions, producing 20–24 kilodalton fragments [31]. In traumatic brain injury, calpain-mediated GFAP-BDP formation predominates over caspase cleavage. GFAP undergoes endogenous citrullination (conversion of arginine residues to citrulline) at multiple sites (R30, R36, R270, R406, R416), potentially altering protein conformation and evoking autoimmune responses [30].

Citrullinated proteins are potent autoantigens implicated in autoimmune diseases such as rheumatoid arthritis. Differential acetylation of lysine residues (K89, K153, K189, K218, K259, K331) has been identified in the spinal cord of amyotrophic lateral sclerosis; the effects on GFAP structure and function remain unclear [28,30,32]. Following CNS injury (traumatic, ischemic, inflammatory, infectious), astrocytes undergo “reactive astrogliosis,” a process involving morphological transformation, proliferation, and altered gene expression. Reactive astrocytes display hypertrophy and enhanced GFAP expression. This process is initiated within minutes to hours of injury and peaks within 24–72 h [17,33].

The mechanism of astrocytic activation involves several pathways. Damage-Associated Molecular Patterns (DAMPs): CNS injury releases intracellular contents (ATP, high-mobility group box 1 [HMGB1], DNA) that activate pattern recognition receptors on astrocytes and microglia [34]. Cytokine Signaling: IL-6, TNF-α, and IL-1β produced by activated microglia and damaged neurons activate astrocytes through cytokine receptors. Excessive glutamate release and activation of ionotropic glutamate receptors (NMDA, AMPA receptors) on astrocytes promote calcium influx and activation. LPS from Gram-negative bacteria activates TLR4 on astrocytes, triggering robust GFAP upregulation and neuro-inflammation.

### 2.3. Release of GFAP into Biofluids Following CNS Injury

GFAP enters the CSF from the brain parenchyma and subsequently reaches the systemic circulation. The kinetics of GFAP release vary by condition: in TBI, GFAP levels peak in blood approximately 20 h post-injury with sustained elevation; in intracerebral hemorrhage, release occurs within 3–4 h; in ischemic stroke, release is delayed (24–48 h post-onset) [35]. The detection of GFAP in blood and CSF following CNS injury may occur through several mechanisms rather than through astrocyte death alone. Traumatic, ischemic, and infectious injuries compromise BBB integrity via disruption of tight junctions (claudins, occludin, zonula occludens proteins) [33]. This allows for leakage of brain-derived proteins, including GFAP, from the CNS parenchyma into the circulation. In severe injury, astrocytic apoptosis or necrosis may contribute to passive release of intracellular GFAP, but circulating concentrations may also reflect reactive astrogliosis, altered barrier permeability, extracellular vesicle-mediated release, CSF drainage, glymphatic clearance, and systemic elimination [33,36,37,38]. The glymphatic system, a waste-clearance pathway involving aquaporin-4 water channels in astrocytic end-feet, facilitates the bulk flow of interstitial fluid containing brain-derived proteins into the CSF and perivascular spaces, which then drain into cervical lymphatics and eventually the systemic circulation [33].

Despite its release secondary to reactive astrogliosis, it is important to distinguish intracellular GFAP expression from circulating GFAP. This differentiation may be achieved through several approaches, which may include analysis of proteolytic proteoforms, clearance kinetics, and integration with other neurovascular co-markers [31]. Under physiological conditions, circulating GFAP represents homeostatic glymphatic turnover or minor microvascular leak. Conversely, acute mechanical or ischemic trauma activates intra-astrocytic calpains and caspases, cleaving the intact 50 kDa intermediate filament into distinct 18–44 kDa GFAP breakdown products (GFAP-BDPs) [35,39]. Furthermore, the crossing of GFAP-BDPs across the compromised BBB leads to a rapid and transient spike in peripheral blood concentration that peaks at approximately 20 h post-injury before its excretion via the kidney [40]. Several studies have also suggested the simultaneous use of GFAP-BDPs with CSF-to-serum gradients or multiplex panels. For instance, given the synchronized elevation of GFAP-BDPs alongside intra-neuronal Ubiquitin C-terminal hydrolase L1 (UCH-L1) or astrocytic S100B following TBI, these co-markers may be used in conjunction to better estimate acute structural neurovascular disruption rather than baseline physiological turnover [40,41] (details will be discussed in subsequent sections).

### 2.4. Breakdown Products as Markers of Astrocyte Injury

GFAP-BDP formation via calpain- or caspase-mediated proteolysis may provide a more injury-associated molecular signal than total GFAP, particularly in settings of severe astrocytic structural disruption. Unlike intact GFAP monomers, which form functional filaments, GFAP-BDP cannot assemble into filaments and may reflect cytoskeletal fragmentation. Furthermore, some evidence suggests that GFAP-BDP may be more closely associated with severe astrocytic structural injury than intact GFAP, whereas intact circulating GFAP may reflect astrocytic activation, BBB disruption, altered drainage, or cell injury depending on pathophysiological context [33,38,42]. However, most clinically available assays measure total GFAP (intact plus cleaved forms), and the differential utility of GFAP versus GFAP-BDP measurement remains an important area for future research [43] (Table 1).

## 3. Clinical Applications in Emergency Medicine

It is noteworthy that the clinical applications of GFAP should be interpreted in light of evidence maturity. At present, mild TBI CT decision-making is the only established emergency medicine application supported by regulatory clearance and prospective validation. Other indications, including stroke differentiation, HIE/cardiac arrest, CNS infections, and sepsis-associated encephalopathy, remain investigational or adjunctive.

### 3.1. Traumatic Brain Injury (TBI)

Multiple clinical studies show that serum GFAP rises after TBI and is associated with the presence and severity of CT-detectable intracranial lesions. Levels are generally higher with more severe, mass-occupying lesions that require neurosurgical intervention, and this relationship has been reported across age groups [44]. GFAP can be detectable within minutes to hours after injury, and early sampling (including prehospital samples) demonstrates that GFAP concentrations correlate with CT lesions, need for neurosurgery, and short-term clinical outcomes [45]. In prospective ED settings, GFAP—especially when treated as a continuous (quantitative) variable—adds diagnostic value to clinical decision rules (e.g., Canadian CT Head Rule, New Orleans Criteria), and in some studies, GFAP alone performed as well as the combination GFAP + UCH-L1 [45]. These findings support GFAP as a potential adjunct to clinical assessment for early triage and for selecting patients for CT or neurosurgical referral, though how best to operationalize thresholds remains unsettled [44,45].

### 3.2. Intracerebral Hemorrhage (ICH) and Ischemic Stroke

GFAP shows a distinct temporal and magnitude pattern between ICH and ischemic stroke. In ICH, rapid astroglial destruction from hematoma expansion produces large, early increases in plasma GFAP; GFAP level correlates with ICH volume and can reach markedly high concentrations shortly after symptom onset. By contrast, GFAP release in ischemic stroke is typically slower and smaller because it follows delayed necrosis and cytolysis [46]. These differences suggest potential utility as an adjunctive marker to aid differentiation of ICH from ischemic stroke in the ED, which might be valuable when the availability of immediate neuroimaging is limited.

### 3.3. Hypoxic–Ischemic Encephalopathy/Cardiac Arrest

GFAP, together with other markers (e.g., tau), has been studied in the context of cardiac arrest and HIE. GFAP and other related astroglial markers in blood may reflect astrocyte-related CNS stress, structural disruption, and BBB dysfunction after global ischemia. Serial measurements can show temporal patterns that relate to injury severity and outcome, but available data are more extensive for other markers (e.g., tau) and for CSF than for GFAP [22,39,47]. At present, the use of GFAP remains exploratory in this setting.

### 3.4. CNS Infections and Other Neurological Emergencies

Most common CNS disorders and classic “stroke mimics” such as migraine or epilepsy do not consistently produce large GFAP increases, supporting reasonable specificity for acute destructive processes [46]. However, GFAP may be elevated when infections cause direct parenchymal injury (e.g., severe bacterial meningitis with encephalitic involvement) or in some tumors (particularly high-grade gliomas) where astrocyte necrosis and BBB disruption occur [44,46]. Thus, while GFAP is informative for acute structural injury, clinicians should interpret elevated values in the full clinical context to avoid misclassification.

### 3.5. Pediatric Considerations

Pediatric data are limited and heterogeneous. Where pediatric studies compared CT-positive versus CT-negative cases, results resembled adult patterns [44]. However, pediatric GFAP ranges have been narrower in the limited studies available, and normative values may differ from adults; therefore, pediatric-specific cutoffs and validation are required before routine ED use in children [44].

### 3.6. Impact on Clinical Capability and Resource Utilization

Beyond diagnostic accuracy, the primary value of GFAP in the emergency department lies in patient disposition. A Negative Predictive Value (NPV) approaching 98.5% for intracranial lesions indicates that GFAP allows clinicians to confidently ‘rule out’ the need for imaging in mild TBI [43,48]. This has a direct impact on reducing ED crowding, decreasing wait times for CT suites, and minimizing the observation time for low-risk patients.

## 4. Diagnostic Accuracy and Operational Considerations

In emergency medicine, the practical relevance of any biomarker is determined by two factors: the precision in identifying the relevant pathology and the integration into time-critical ED workflows [22]. GFAP stands out because circulating concentrations may reflect an astrocytic-related CNS pathology, particularly when interpreted alongside clinical findings, imaging, sampling timing, and adjunct biomarkers. Evidence suggests that GFAP can rise within the first hour after TBI, and it is diagnostically useful when measured within 30–60 min of injury. GFAP demonstrated strong discrimination for CT-detectable intracranial lesions when measured within 30 min (AUC 0.88, 95% CI 0.85–0.92) in a large multicenter cohort of suspected moderate-to-severe TBI who underwent very early sampling [22]. Its performance remained similarly high when measured within 60 min (AUC 0.89, 95% CI 0.86–0.92), outperforming UCH-L1 and MAP-2 at both timepoints [22].

In sepsis-associated encephalopathy (SAE), since a standardized cut-off point has not yet been defined, the key question is whether GFAP provides a biological signal of astroglial involvement in SAE. Recent evidence showed that serum GFAP levels are significantly higher in SAE compared to septic patients without encephalopathy (pooled effect size SMD 3.63, with high heterogeneity and a small number of contributing GFAP studies), suggesting a measurable astroglial-associated signal of CNS involvement [22]. At the same time, GFAP did not exhibit consistent prognostic differentiation between survivors and non-survivors, limiting how far it can be pushed for mortality prediction. Nevertheless, the current evidence remains limited by heterogeneity, small sample sizes, and a lack of standardized cutoffs.

In hemorrhagic cases, GFAP’s performance becomes frequently clinically important since it can increase significantly with bleeding-related astrocytic damage. In subarachnoid hemorrhage, one study reported strong ROC performance using GFAP in both CSF and serum (AUC 0.893 in CSF and 0.820 in serum), with proposed cutoffs that achieved high sensitivity and specificity in their cohort (for example, a CSF cutoff around 0.316 ng/mL showed sensitivity 89% and specificity 82%, while a serum cutoff around 0.071 ng/mL showed sensitivity 74% and specificity 89%). These findings show that GFAP may provide benefit when early imaging is equivocal or when physicians require additional assurance about the hemorrhagic condition and prognosis [15].

Confounding factors include factors like age, baseline GFAP levels, which can be higher in older adults due to chronic neuroinflammation, and renal function, as GFAP is cleared by the kidneys; patients with end-stage renal disease (ESRD) might have falsely elevated baselines. Also, chronic disease patients with Multiple Sclerosis or Alzheimer’s will have higher levels, which may complicate acute triage [46].

## 5. Overview and Comparison of Major CNS Biomarkers (S100B, UCH-L1, tau, NSE)

GFAP is more specific to astrocytes in the CNS and is rarely detectable in peripheral blood unless CNS injury occurs [47]. In contrast, S100B is produced not only by astroglia but also by extracerebral tissues (e.g., adipocytes, muscle, and chondrocytes), reducing its CNS specificity, neuron-specific enolase (NSE) is a neuronal glycolytic enzyme that lacks strict brain specificity and is strongly influenced by hemolysis [49], tau is a neuron-specific microtubule-associated axonal protein, and ubiquitin C-terminal hydrolase L1 (UCH-L1) is a neuron-specific enzyme reflecting neuronal and axonal damage [50]. This biological distinction is reflected clinically (Table 2).

In the bloodstream, GFAP exhibits favorable kinetics, with detectable elevations appearing within approximately 1 h after CNS injury, peaking around 20 h, and remaining elevated for days (half-life ~24–48 h). UCH-L1 also has similar kinetics. By contrast, tau levels typically rise more slowly, reflecting delayed axonal injury rather than immediate tissue disruption [49]. NSE concentrations are more variable and can be falsely elevated due to hemolysis or extracerebral sources, limiting interpretability in acute settings [49]. In contrast, S100B shows a rapid, but transient increase (half-life ~0.5–2 h, peak < 6 h) [50].

The diagnostic significance of GFAP is most clearly established in TBI. Blood GFAP demonstrates strong diagnostic accuracy for identifying intracranial lesions and, together with UCH-L1, has received FDA authorization to support CT decision-making in patients with mild TBI [15,50]. However, comparative ED studies demonstrated that GFAP alone consistently provides a stronger and more reliable signal, and that combining it with UCH-L1 does not significantly improve diagnostic performance beyond GFAP alone. GFAP correlates closely with CT- and MRI-positive brain injury and consistently outperforms NSE and UCH-L1 for detecting structural lesions, particularly in the hyperacute phase [15]. Tau contributes less to early diagnostic decision-making, as it primarily reflects axonal injury that evolves over time rather than immediate structural damage [50]. Beyond trauma, GFAP also shows strong diagnostic potential in sepsis-associated encephalopathy, where GFAP levels are significantly higher in patients with neurological involvement and correlate with disease severity [49,51]. Meta-analytic evidence demonstrates substantially larger effect sizes for GFAP than tau in differentiating septic patients with versus without encephalopathy [51]. However, NSE, UCH-L1, Tau, and S100β have significant results regarding distinguishing survivors from non-survivors in this context [49,51].

From a prognostic perspective, elevated GFAP levels generally reflect more severe CNS injury and worse outcomes. In acute TBI, higher GFAP concentrations are associated with increased likelihood of neurosurgical intervention and early mortality [15]. In subarachnoid hemorrhage, elevated GFAP correlates with greater injury burden and poorer neurological outcomes [36]. In chronic neurological and neurodegenerative conditions, blood GFAP levels are associated with progressive CNS damage and worsening disability [47,51]. While tau biomarkers provide prognostic information regarding long-term neuronal degeneration and axonal pathology, NSE may offer prognostic insight in selected systemic conditions (e.g., sepsis). Across acute and chronic neurological conditions, GFAP remains a highly informative marker for broad prognostic assessment [47], though its clinical dependability must be interpreted alongside the physiological confounders and assay variations discussed throughout this review.

## 6. Implementation Challenges

### 6.1. Standard Levels

GFAP is a marker of CNS injury, as it signals astrocytic activation following injury [14,54]. Considering the proximity of cerebrospinal fluid (CSF) to the brain parenchyma, GFAP concentrations in the CSF tend to be significantly higher than in blood, and in some cases it exceeds 100 times higher levels in CSF compared to blood levels. Short-term fluctuations in GFAP levels can be detected more easily and quickly in the CSF than in the blood, yet large changes can also be observed in the blood despite its smaller proportional difference in concentration relative to CSF [33,55]. Furthermore, other studies have reported that the correlation between GFAP levels in blood and CSF is only moderate [56,57]. Given the differences in GFAP concentrations between CSF and blood, measuring GFAP in blood has historically been challenging. However, recent advancements in detection technologies have enabled the measurement of low-abundance, brain-derived proteins in blood using conventional enzyme-linked immunosorbent assays (ELISAs) [37,58]. Such advancements have made it possible to detect low-abundance proteins in blood, such as GFAP, using highly sensitive immunoassay platforms, including single-molecule array (Simoa^®^, Billerica, MA, USA) [59], Ella Simple Plex^®^ (Bio-Techne, San Jose, CA, USA) [60], and Meso Scale Discovery^®^ (MSD^®^) (e.g., MESO QuickPlex^®^ SQ) (Meso Scale Diagnostics, LLC, Rockville, MD, USA) [61].

### 6.2. GFAP Immunoassays Across Multiple Platforms

GFAP has been shown to be a reliable and sensitive biomarker for the diagnosis of neurodegenerative diseases, neuroinflammatory disorders, and traumatic brain injury [47,52]. However, there is a lack of a standardized method to quantify GFAP levels across different immunoassay platforms. This lack poses a challenge for the clinical implementation of GFAP [55,62]. While Simoa^®^ is a widely recognized technology known as the gold standard for ultra-sensitive neuro-biomarker quantification in research and specialized laboratory settings, its implementation in routine clinical laboratories remains limited. Therefore, there is a critical need to adapt and validate GFAP assays on conventional, widely available immunoassay platforms to ensure consistent results. Currently, there are many commercially available immunoassay instruments based on sandwich immunoassay principles; however, the absence of reference materials, antibody variations, and different calibrators, along with instrument-specific characteristics, poses challenges for standardizing result interpretation across laboratories using different methodologies [54].

## 7. Clinical Implementation

Recent prospective ED and prehospital cohorts demonstrate that GFAP can be measured very early after injury and can meaningfully stratify risk and CT positivity [15,45]. In practical terms, GFAP shows promise as an adjunct to clinical decision rules to reduce unnecessary CT imaging and to support triage, especially when the clinical exam is unreliable. In some studies, GFAP alone added most of the biomarker benefit compared with combined GFAP + UCH-L1 testing [45].

Exploratory thresholds from early cohorts illustrate possible rule-out and rule-in cutoffs (e.g., <30 pg/mL for high-sensitivity rule-out vs. values in the thousands of pg/mL for rule-in of CT lesions or neurosurgical need), but these thresholds are assay-dependent and require prospective validation in different ED populations before routine use [45].

To move GFAP safely into ED practice, standardized assay calibration, agreed-upon units and reporting, time-window-standardized sampling, and large multicenter diagnostic accuracy studies that report sensitivity, specificity, and effects on patient management and outcomes are needed [44,45]. Future research may focus on the role of GFAP in rare congenital central nervous system disorders, for instance, Meckel–Gruber syndrome associated with Dandy–Walker malformation, which can present with severe neonatal neurological complications requiring emergency evaluation [47,63].

## 8. Future Direction

Future research should aim to determine whether GFAP, GFAP-BDPs, or both provide clinically actionable information in particular emergency syndromes. The key question is not simply whether GFAP correlates with neurological injury severity, but whether it can independently identify or safely rule out a prespecified condition or instead provide incremental value when combined with clinical examination, validated scores, and imaging. This distinction is important because commercial immunoassays may detect intact GFAP and/or fragments according to their antibody epitopes, while proteolysis, post-translational modification, aggregation, and sample handling may affect the concentration reported as “GFAP.” Future analytical studies should therefore characterize the molecular species being measured and establish concordance across serum, plasma, and whole-blood assays [31,64,65].

In acute ischemic and hemorrhagic stroke, the most plausible near-term application is the use of GFAP as an adjunct for estimating the probability of intracranial hemorrhage and supporting prehospital or emergency-department triage, without delaying definitive neuroimaging or reperfusion pathways [62]. Prospective studies should enroll consecutive patients with suspected stroke, including ischemic stroke, ICH, transient ischemic attack (TIA), and common stroke mimics. They should prespecify the sampling time, assay, threshold, acceptable false-negative rate, and intended clinical decision. Although recent studies suggest that age- and sex-dependent thresholds and serial measurements may improve ICH rule-out, these approaches require external validation across populations, transport systems, assays, and clinically relevant disease prevalence [66]. Research should also determine whether GFAP changes management—for example, transport destination, preparation for neurocritical or neurosurgical care, prehospital blood pressure management, or allocation of mobile stroke unit resources—and measure effects on treatment times, safety, imaging use, disposition, costs, equity, and patient-centered outcomes [38,42,62,66].

In TBI, GFAP and GFAP-BDPs have demonstrated value for identifying CT-visible intracranial injury, particularly in mild TBI, and GFAP-containing laboratory and point-of-care tests now provide a practical basis for selected emergency-department CT-triage applications [15,64,67]. Future studies should assess their performance in underrepresented groups, including older adults, children, patients receiving anticoagulants or antiplatelet therapy, and patients with polytrauma, hemorrhagic shock, delayed presentation, or repeated head injury. Biomarker-guided pathways should be compared with existing clinical decision rules, with outcomes including missed clinically important injuries, unnecessary CT scans, observation time, return visits, resource use, and cost. Serial GFAP measurements also warrant investigation for detecting evolving secondary injury, neurological deterioration, and the need for repeat imaging or higher-level monitoring. Prognostic studies should evaluate functional recovery, persistent post-concussive symptoms, cognition, and quality of life rather than CT findings alone [15,67,68].

GFAP-BDPs and other proteoforms represent an important molecular research direction. Early studies associated plasma GFAP-BDPs with CT lesions and injury severity, while recent proteomic work has identified TBI-associated fragments and post-translational modifications, some of which may be more closely related to long-term outcomes than uncleaved GFAP [31]. Future work should determine whether specific fragments distinguish focal hemorrhage from diffuse injury, whether fragment-to-full-length ratios improve diagnosis or prognosis, and whether proteoform profiles identify ongoing proteolysis, secondary injury, persistent post-traumatic symptoms, or post-traumatic neurodegeneration [64].

Across stroke, TBI, coma, and other neurological emergencies, prognostic models should distinguish association from clinical prediction by assessing calibration, incremental value over clinical and imaging predictors, temporal reproducibility, decision-curve net benefit, and the consequences of acting on the result. Point-of-care feasibility is increasingly supported in selected TBI and coma settings, but broader implementation will require assay harmonization, robust sample processing, quality control, cost-effectiveness evaluation, equitable performance, and prospective impact trials [67]. Until these questions are answered, GFAP and its molecular derivatives are best regarded as condition-specific adjuncts that refine pretest probability and characterize tissue injury within existing clinical and imaging pathways, rather than as universal standalone diagnostic or rule-out tests [15,31,62].

## 9. Conclusions

The current early evidence suggests that GFAP is a promising astrocyte-enriched biomarker that may help address a critical gap in the rapid, objective assessment of CNS injuries. The clinical value of its use is mainly demonstrated in the triage of TBI, where its rapid kinetics—rising within 1 h and peaking around 20 h—facilitates early identification of intracranial lesions. GFAP is an FDA-cleared biomarker for mild TBI that aids ED physicians in determining when CT scans are required, minimizing unnecessary radiation exposure and better utilizing hospital resources. Beyond trauma, growing evidence shows a promising diagnostic role of GFAP to distinguish hemorrhagic from ischemic strokes in time-sensitive windows, and may serve as a biological marker of CNS involvement in SAE.

Ultimately, GFAP might move emergency neurology from an observational ‘wait and see’ model to a precision-based ‘measure and act’ model that ensures the proper patients receive advanced imaging while the ‘worried well’ are safely sent home.

From a prognostic perspective, elevated GFAP concentrations may be associated with injury severity, the need for neurosurgical intervention, and long-term disability across various acute and chronic conditions. Current evidence indicates that GFAP is frequently superior to other markers such as UCH-L1 and NSE in the hyperacute phase; however, its full integration into routine emergency practice still faces hurdles. Challenges include a lack of standardization across different immunoassay platforms, the need to validate assay-specific thresholds, and insufficient awareness among front-line clinicians.

## Figures and Tables

**Table 1:** Comparison between GFAP and GFAP-BDP.

Feature	Intact GFAP	GFAP-BDP (Breakdown Products)
Mechanism of Formation	Natural synthesis; baseline structural protein.	Produced via calpain proteolysis during injury.
Structural Function	Assembles into functional filaments (astrocyte “skeleton”).	Cannot assemble into filaments; represents structural disintegration.
Primary Pathological Signal	Indicates astrocytic activation and Blood–Brain Barrier (BBB) disruption.	May be associated with severe astrocytic structural disruption, including necrosis or apoptosis in some contexts.
Diagnostic Specificity	Lower; elevated by both reactive astrogliosis and cell death.	Potentially higher for severe injury, although interpretation depends on assay specificity and clinical context.
Clinical Interpretation	Reflects a “stressed” or reactive brain state.	May provide a more injury-associated signal than intact GFAP but does not independently prove irreversible cellular damage.

**Table 2:** Comparison of Major CNS Biomarkers.

Biomarker	Cell Source	Kinetics	Evidence-Based Strengths	Evidence-Based Limitations
GFAP	Astrocyte-enriched protein; rarely detectable in blood without CNS injury [47]	Rises ~1 h, peaks ~20 h, half-life 24–48 h	FDA-cleared (with UCH-L1) for CT decision-making in mild TBI [22,50] Emerging/investigational diagnostic values for stroke, SAE, HIE, SAH Outperforms NSE and UCH-L1 for structural lesions, especially hyperacute phase [22,50] Elevated in SAE, correlating with severity; larger effect size than tau [49,51] Correlates with injury burden/outcome in SAH [36] Most reliable single marker for prognosis across acute and chronic CNS conditions [47]	FDA clearance pathway requires pairing with UCH-L1 rather than stand-alone use [22,50] Reflects astrocytic reactivity, which may lag the moment of primary neuronal injury
Serum UCH-L1 [50,52]	Neuron-specific enzyme reflecting neuronal/axonal damage	Rises < 1 h, peaks 9 h, half-life 7–9 h	Co-cleared by FDA with GFAP for CT decision-making in mild TBI [21,45]	Adds little diagnostic value beyond GFAP alone in comparative ED studies [22] Combining with GFAP does not significantly improve performance over GFAP alone [22]
S100B	Astroglia + extracerebral (adipocytes, muscle, chondrocytes)	Very rapid, transient: peak < 6 h, half-life 0.5–2 h	Distinguishes sepsis survivors from non-survivors [44,46]	Low CNS specificity Confounded by extracerebral tissue damage [47]
NSE [53]	Neuronal glycolytic enzyme, not brain-specific	Rises 2–6 h, peaks 24–48 h, half-life ~24–30 h	Offers prognostic insight in selected systemic conditions (e.g., sepsis survival) [49,51]	Strongly and falsely elevated by hemolysis Variable kinetics limit interpretability in acute settings [49]
Tau [20,50]	Neuron-specific axonal microtubule-associated protein	Rises slowly 1–6 h, reflecting evolving axonal injury peaks 12–24 h, half-life ~10 h	Prognostic value for long-term neuronal degeneration/axonal pathology [47,51]	Contributes little to early/acute diagnostic decisions—reflects delayed rather than immediate injury [50] Smaller effect size than GFAP for differentiating septic encephalopathy [51]

## Data Availability

Data are available upon reasonable request to the corresponding author.
